# Treatment of Rifampicin-Resistant Tuberculosis Disease and Infection in Children: Key Updates, Challenges and Opportunities

**DOI:** 10.3390/pathogens11040381

**Published:** 2022-03-23

**Authors:** Pauline Howell, Jay Achar, G. Khai Lin Huang, Andrei Mariandyshev, H. Simon Schaaf, Anthony J. Garcia-Prats

**Affiliations:** 1 Clinical HIV Research Unit, Department of Internal Medicine, Faculty of Medicine and Health Sciences, University of Witwatersrand, Sizwe Tropical Disease Hospital, 2 Modderfontein Road, Sandringham, Johannesburg 2131, South Africa; 2 Department of Global Public Health, Karolinska Institutet, 171 77 Stockholm, Sweden; jay.achar@ki.se; 3 Tuberculosis Elimination and Implementation Science Working Group, Burnet Institute, 85 Commercial Road, Melbourne, VIC 3004, Australia; khai.huang@burnet.edu.au; 4 Department of Phthisiopulmonary, Northern State Medical University, Troitsky Avenue 51, Arkhangel’sk 163000, Russia; maryandyshev@gmail.com; 5 Scientific Department, Northern Arctic Federal University, Naberezhnaya Severnoy Dviny 17, Arkhangel’sk 163002, Russia; 6 Desmond Tutu TB Centre, Department of Paediatrics and Child Health, Faculty of Medicine and Health Sciences, Stellenbosch University, P.O. Box 241, Cape Town 8000, South Africa; hss@sun.ac.za (H.S.S.); garciaprats@wisc.edu (A.J.G.-P.); 7 Department of Pediatrics, University of Wisconsin School of Medicine and Public Health, 2870 University Avenue, Madison, WI 53705, USA

**Keywords:** tuberculosis, rifampicin-resistant, multidrug-resistant, treatment, prevention, paediatrics, children

## Abstract

Children affected by rifampicin-resistant tuberculosis (RR-TB; TB resistant to at least rifampicin) are a neglected group. Each year an estimated 25,000–30,000 children develop RR-TB disease globally. Improving case detection and treatment initiation is a priority since RR-TB disease is underdiagnosed and undertreated. Untreated paediatric TB has particularly high morbidity and mortality. However, children receiving TB treatment, including for RR-TB, respond well. RR-TB treatment remains a challenge for children, their caregivers and TB programmes, requiring treatment regimens of up to 18 months in duration, often associated with severe and long-term adverse effects. Shorter, safer, effective child-friendly regimens for RR-TB are needed. Preventing progression to disease following *Mycobacterium tuberculosis* infection is another key component of TB control. The last few years have seen exciting advances. In this article, we highlight key elements of paediatric RR-TB case detection and recent updates, ongoing challenges and forthcoming advances in the treatment of RR-TB disease and infection in children and adolescents. The global TB community must continue to advocate for more and faster research in children on novel and repurposed TB drugs and regimens and increase investments in scaling-up effective approaches, to ensure an equitable response that prioritises the needs of this vulnerable population.

## 1. Introduction

Globally, there are an estimated 2 million children infected with rifampicin-resistant tuberculosis (RR-TB; TB resistant to at least rifampicin) or multidrug-resistant TB (MDR-TB; RR-TB with confirmed resistance to isoniazid), and each year an estimated 25,000–30,000 children develop RR-TB disease [1,2,3]. TB has high morbidity and mortality when untreated in children, with an estimated 96% of all childhood deaths from TB overall occurring in those not receiving treatment, with 86% occurring in children under 5 years of age [4]. However, when treated appropriately, children generally respond well, including to RR-TB treatment. Overall treatment success was reported in 78% of children with RR-TB in a 2018 systematic review and individual patient data meta-analysis, with some studies reporting greater than 90% treatment success [5]. This is substantially better than in comparable adult RR-TB cohorts, where 50–60% of patients are successfully treated in programmatic settings [2]. Despite this, RR-TB treatment remains difficult both for children, their caregivers and TB programmes, requiring treatment regimens of up to 18 months in duration, often associated with severe and long-term adverse effects. For children, the limited availability of child-friendly formulations is an additional barrier. Shorter, safer, effective and child-friendly regimens for RR-TB are needed. In addition, RR-TB disease is underdiagnosed and undertreated and improving paediatric case detection and appropriate treatment initiation is a priority.

Preventing disease following *Mycobacterium tuberculosis* infection is a key component of TB control. Systematic screening of contacts and offering preventive treatment for those at risk is a World Health Organization (WHO) priority for reducing TB incidence, morbidity, mortality and costs [6]. The lack of high-quality evidence for approaches to treating MDR-TB infection has been a barrier to scaling-up this important intervention. In 2018, a United Nations (UN) high-level meeting on TB committed countries to provide preventive treatment to at least 30 million at-risk people, including 4 million children, between 2018 and 2022 [7]; however, only one-third of household contacts under 5 years old, who were eligible for TB preventive treatment (TPT) in 2019 received it [2], and even fewer children received preventive treatment for MDR-TB infection.

The last few years have seen exciting advances in the treatment of RR-TB infection and disease. In this article, we highlight recent updates, ongoing challenges and forthcoming advances in the treatment of RR-TB disease and infection in children and adolescents. We also briefly discuss key issues in case detection of RR-TB.

## 2. RR-TB Case Detection 

The difficulty in attaining source case information, variations in clinical presentation [8] and non-specific radiological changes [9] complicate TB detection in children and contribute to misdiagnosis, incorrect treatment, and ultimately TB-related mortality [10]. Approximately 30% of paediatric cases with pulmonary TB are bacteriologically confirmed [11], resulting in a reduced detection of drug resistance and a greater emphasis on clinical judgement to make a diagnosis and initiate treatment for RR-TB. Paediatric TB is underdiagnosed, with an increasing gap with decreasing age. The WHO estimates that up to 65% of children under 5 years with TB disease remain undiagnosed [2]. Since the detection of RR-TB requires additional expertise and can require access to more advanced diagnostic tests, the detection gap for these children is even wider. In 2018, 3398 children were reported to have started treatment for RR-TB globally, representing 2.2% of all TB treatment initiations. In 2019, this rose to 5588 (3.2%) before falling in 2020 to 3235 (2.5%), primarily due to the effects of the COVID-19 pandemic [2]. Notifications remain far below both the estimated actual number of children with RR-TB disease and the stated global target of treating 115,000 children for RR-TB between 2018 and 2022 [7].

The COVID-19 pandemic has worsened existing challenges with the detection and treatment of both TB disease and infection. For the first time in more than a decade, TB mortality has increased [2]. Modelling studies predict that over the next 5 years there will be as many as 6.3 million COVID-related excess incident TB cases globally, representing a 10.7% increase, and as many as 1.3 million, or up to 20% excess deaths from TB [12,13]. The 2021 WHO Global TB Report confirmed that from 2019 to 2020 there was an 18% reduction in notified TB cases globally, a 21% reduction in persons receiving treatment for TB infection, and an increase in TB deaths to 1.5 million, up from 1.4 million [2]. There was also a 22% decrease in RR-TB cases detected overall, with a larger decrease in the paediatric group. Equitable and high COVID-19 vaccine coverage are required to enable countries to rebuild their TB services. Using existing COVID-19 cases platforms, the worsening TB pandemic should be highlighted to hold governments accountable and advocate for increased funding. COVID-19 advances can be employed to improve case detection and create novel TB tools [14].

Strategies to improve RR-TB case detection must be urgently implemented. Updated recommendations for RR-TB case finding activities include the provision of decentralised, family-centred, integrated services in high TB-burden areas that supplement existing services, and clinical training for primary healthcare workers [10,15]. Research is ongoing to identify more sensitive and child-friendly tools and sampling techniques to diagnose TB and drug-resistant TB (DR-TB) in children [10]. These should be scaled up, but the sensitivity of existing diagnostic tests for children with TB remains low and these should not be relied on as the only or even the most important method for increasing RR-TB diagnoses in children. While awaiting newer detection tools, rapid scale-up of the clinical diagnosis of RR-TB must be prioritised. This relies not on new technologies, but on the training of healthcare workers and the development of appropriate policies and guidelines. Evidence-based, implementable treatment decision algorithms appropriate to the setting are needed, particularly for young children, where clinicians are most hesitant; such algorithms are expected from the WHO by the beginning of 2022 [10]. Bi-directional integrated testing of both TB and COVID-19 should be introduced programmatically along with catch up campaigns to find the backlog of missed TB cases [16]. Improved uptake of household contact tracing will identify more cases of RR-TB disease and infection in children.

Greatly improved identification of children with RR-TB disease and infection is needed to maximise the impact of improved treatment tools.

## 3. Treatment of RR-TB Disease

There have been many recent exciting developments in the treatment of RR-TB disease. Shorter, injectable-sparing regimens utilising new and repurposed TB drugs are being evaluated in adult trials. Efficacy of these regimens can reasonably be extrapolated to children without conducting large paediatric efficacy trials, but only if the dosing and safety of the individual drugs are known in children, and child-friendly formulations are available. Thus, an understanding of the rapidly evolving adult trial landscape is critical for prioritising paediatric research needs.

### 3.1. Updates on the Overall (Adult) RR-TB Treatment Landscape

In 2016, observational studies and data from the STREAM trial [17] supported the WHO decision to conditionally approve a 9–12-month regimen for selected patients with RR-/MDR-TB [18]. In 2019, a 6-month regimen consisting of bedaquiline, pretomanid and linezolid (BPaL) for patients meeting particular inclusion criteria, was approved by the US Food and Drug Administration [19] based on the Nix-TB trial results [20] and is now being evaluated in programmatic settings under operational research conditions 4141. In 2019, the WHO recommended an all-oral RR-TB regimen for all patients [18]. This included prioritising newer drugs, such as bedaquiline and delamanid, and repurposed drugs, such as linezolid and clofazimine. In addition, ototoxic second-line injectable drugs were removed from most treatment regimens. 

Ongoing or recently completed RR-TB treatment clinical trials evaluating different short, all-oral regimens, most with combinations of the key drugs bedaquiline, delamanid, pretomanid, clofazimine, linezolid, moxifloxacin, and levofloxacin (Table 1), offer the prospect of broader recommendations for 6-month regimens. The ZeNix trial confirmed the efficacy of a 6-month regimen containing bedaquiline, pretomanid, and linezolid (BPaL) while establishing the lowest dose and shortest duration of linezolid to limit toxicity. The trial confirmed non-inferiority at lower doses and durations of linezolid as compared to BPaL in the Nix-TB trial, with the added benefit of fewer adverse events [21]. A trial evaluating 6-month regimens for RR-TB with and without additional resistance to fluoroquinolone drugs, TB-PRACTECAL [22], concluded that a regimen containing pretomanid, bedaquiline, linezolid and moxifloxacin was 89% efficacious with fewer adverse effects than conventional treatment [23]. 

Although still longer and more toxic than desired, RR-TB treatment is becoming more acceptable and tolerable for affected adults, but many of the newest regimens remain out of reach for children until critical paediatric research gaps are addressed. 

### 3.2. Updated Pharmacokinetic and Safety Data in Children

A priority for improving the treatment of RR-TB disease in children is ensuring adequate pharmacokinetic (PK) and safety data to allow the extrapolation of emerging efficacy data from adult trials [26,27,28]. The last few years have seen substantial PK, dosing and safety data for key repurposed second-line TB drugs in children, including levofloxacin [29,30,31,32,33], moxifloxacin [33,34] and linezolid [28], that have substantially improved the evidence base for dosing recommendations in young children for these important drugs [35]. These data and current paediatric dosing recommendations for children are summarised in Table 2.

The most exciting emerging data is related to the new TB drugs, bedaquiline and delamanid. In addition to early safety and effectiveness data in children and adolescents from off-label or compassionate use of bedaquiline and delamanid, paediatric trial results are now becoming available [26,27]. The C211 paediatric bedaquiline phase I/II trial, which is using a traditional age de-escalation study design, has completed its first two cohorts, which led to the recent United States Food and Drug Administration (FDA) approval for bedaquiline down to age 5 years [39]. The P1108 (NCT02906007) paediatric phase I/II trial has also shared emerging data on bedaquiline in children down to age 0 years [40]. Paediatric data on delamanid from Otsuka’s 232 and 233 phase I/II trials have led to recent approval by the European Medicines Agency (EMA) of delamanid for children ≥10 kg body weight [41]. The optimal dose for children below this weight is being evaluated in the IMPAACT 2005 study (NCT03141060). During 2021, the WHO re-evaluated data on the use of bedaquiline and delamanid in children and has recommended the use of both drugs in children of all ages for RR-TB [10]. Although small numbers of children were included, safety was similar to adult cohorts and population PK models derived from data from study participants are informing new dosing recommendations for children of all ages [10]. Since bedaquiline has become a key drug in all regimens recommended for the treatment of RR-TB, the expansion of its approval to all children is a particularly important milestone. 

Despite these exciting advances, important knowledge gaps remain. Clofazimine has been used in children for the treatment of leprosy, but there is limited data about its PK, optimal dosing, drug-drug interactions and safety in children. It is being used regularly in children in the WHO 9–11-month RR-TB regimen and as a Group B drug in individually constructed regimens, but an improved paediatric evidence base is needed, and will be informed by two ongoing paediatric trials. Pretomanid has no paediatric safety or PK data available and is therefore not currently approved for use in children. A single-dose study in children is planned to start in 2022 (IMPAACT 2034) and multi-dose trials are contingent on this and additional safety data in adults [42]. 

Paediatric dose optimisation for newer drugs is also required. Simpler dosing regimens for BDQ (200 mg daily for 8 weeks, followed by 100 mg daily for 16 weeks) have been evaluated in two adult clinical trials [21,42] and were found to be well tolerated. Updates to recommendations for programmatic use and evaluation in children are awaited. An approach of once-daily delamanid dosing is being increasingly utilised and is being studied in children following RR-TB exposure in the PHOENIx trial (NCT03568383); this dosing approach may need confirmatory PK studies in children with RR-TB disease.

### 3.3. Future Regimens for Children

The recent SHINE trial [43] confirmed non-inferiority of 4-month treatment durations for drug-susceptible TB compared to the standard 6-months for children with non-severe TB. This demonstrates what many clinicians have long suspected, that children with non-severe (paucibacillary) disease could be effectively treated with shorter durations than are required in adults (with high bacillary loads in cavitary pulmonary TB). In 2019, the WHO acknowledged in their treatment guidelines update that children with non-severe disease due to RR-TB may be sufficiently treated using shorter treatment durations, but specific actionable recommendations were not made [18]. Thus, evaluating shorter, less-intense regimens for children with RR-TB remains important, but there have been few studies in this area. The pragmatic BEAT-Tuberculosis trial (NCT04062201) compares a 6-month regimen of bedaquiline, delamanid, linezolid and levofloxacin and/or clofazimine (dependant on fluoroquinolone drug susceptibility testing) with the South African national RR-TB standard of care. The study includes opportunistic enrolment of children greater than 6 years and interim results are expected in July 2022. 

There is a growing pipeline of new TB compounds, including a number of compounds entering phase two clinical development in adults [44]. This includes several completely new drug classes as well as compounds in existing classes that may have advantages over existing drugs, such as oxazolidinones, with improved safety and efficacy compared to linezolid. Without intentional action and additional investment in paediatric development of these new compounds, this will almost certainly result in compounding delays in access to better treatments for children than have been observed to date.

### 3.4. Other Considerations 

#### 3.4.1. Child-Friendly Formulations

In recent years, child-friendly second-line TB drug formulations have gradually become more widely available. The Stop TB Partnership’s Global Drug Facility (GDF), the largest global provider of quality-assured TB drugs, has worked to include child-friendly formulations for bedaquiline, clofazimine, cycloserine/terizidone, ethambutol, ethionamide, levofloxacin, moxifloxacin and pyrazinamide in their catalogue, and in partnership with the Sentinel Project [45], to support their uptake globally [46]. While countries remain where these formulations are unapproved and therefore not accessible (e.g., the European Union and South Africa), countries that do have access should prioritise their procurement and incorporate these into child-friendly services. 

A bedaquiline 20 mg dispersible tablet has been approved by the FDA and is available from the GDF. In 2021, the EMA approved a 25 mg dispersible delamanid formulation for use in children weighing over 10 kg [41], which is expected to become available in early 2022. Dispersible formulations of 10mg and 50mg of pretomanid have been developed for the planned paediatric trials [47]. Gaps for formulations include a linezolid dispersible tablet, currently under development by multiple generic pharmaceutical companies, and more palatable formulations of the existing, bitter moxifloxacin dispersible tablets which are being addressed by the Unitaid-funded BENEFIT Kids project [48].

#### 3.4.2. Adolescents

Although the inclusion of adolescents in adult DR-TB trials is an exciting development, low enrolment numbers are limiting the benefit. As an example, the Nix-TB trial allowed the inclusion of participants 14 years or older, but few adolescents were included. The FDA approved the regimen in August 2019 [19] and the EMA in July 2020 [49], but only for adults. With the rollout of programmatic access to the BPaL regimen [50], implementing countries are using their discretion to include adolescents of 14 years and older in line with the Nix-trial design [51]. The ZeNix and TB-PRACTECAL trials also included adolescents over the ages of 14 and 15 years, respectively, but it is unclear how many were enrolled and whether adolescents will be included in any resultant guideline changes.

#### 3.4.3. Novel Approaches

Interesting novel treatments being considered include long-acting or extended-release (LA/ER) formulations for the treatment and prevention of TB. Pre-clinical animal models have shown proof of concept for the treatment of TB infection with LA/ER isoniazid [52], a potentially attractive approach when bundled with HIV treatment.

## 4. Treatment of MDR-TB Infection 

Given their high risk of progression from infection to disease, the WHO recommends that people living with HIV and household contacts under 5 years of age exposed to bacteriologically confirmed pulmonary TB should receive preventive treatment. Full implementation would dramatically reduce incident TB cases and TB-related deaths in children, and remains an urgent priority [53]. However, treatment of MDR-TB infection has been limited by a lack of high-quality data on the efficacy and safety of an appropriate regimen.

Although the WHO has not recommended the systematic treatment of any particular group following MDR-TB exposure, they propose the use of preventive treatment following an individualised risk assessment for high-risk household contacts, such as children under 5 years of age and people living with HIV [54]. In 2019, a joint practice guideline from US and European agencies advocated 6-12 months of treatment with a fluoroquinolone alone or in combination with a second drug, to which the source case’s isolate is susceptible, for people exposed to MDR-TB [55]. 

Trials evaluating the efficacy of treatment of infection with TB resistant to at least rifampicin and isoniazid are ongoing. TB-CHAMP [56] compares 24 weeks of levofloxacin with placebo in children under 5 years of age. The VQUIN trial [57] also compares levofloxacin with placebo amongst adult household contacts of MDR-TB. PHOENIx [58] compares 6 months of delamanid versus the standard-dose of isoniazid amongst MDR-TB household contacts, including children. While results from these trials are not yet available, a systematic review and meta-analysis of five observational studies concluded that preventive treatment may reduce risk by up to 90% [59]. Another modelling study reported that fluoroquinolone-based preventive treatment of infected MDR-TB household contacts would be highly beneficial, even if the efficacy is much lower than 90% [60]. PK, safety and tolerability data for child-friendly levofloxacin formulations [29,31,61] will facilitate the rapid implementation of recommendations following MDR-TB exposure in children that might follow results from VQUIN and TB-CHAMP. 

Widespread programmatic implementation of treatment of children with exposure or infection after contact with MDR-TB will depend on high-quality evidence from the ongoing trials. However, expert opinion consistently favours the use of fluoroquinolone-based regimens in most children for MDR-TB exposure, for whom this is likely to be safe and for whom RR-TB disease and treatment remain a substantial risk [54]. Practical guidance on this is available, and clinicians caring for MDR-TB exposed children should consider implementing this approach, pending more definitive data [62]. 

## 5. Conclusions 

The significant global gap in case finding, diagnosis and appropriate treatment of children with RR-TB require greater uptake of clinical diagnosis, improved diagnostic tests, and better household contact tracing activities. The TB community must redouble its commitment to case detection and look for synergies with COVID-19 case detection strategies.

Emerging PK and safety data supporting the use of newer second-line TB drugs in all children will allow children to benefit from the growing evidence supporting shorter, safer, effective treatment for RR-TB disease. Addressing remaining knowledge gaps and more efficient paediatric evaluation of new TB compounds are important ongoing priorities. Treatment shortening and simplification will ultimately lead to greater access for all children and adolescents. Anticipated results from trials of strategies to treat MDR-TB infection in high-risk infected or exposed children will have large public health implications.

The latest WHO guidelines [63] and operational handbook [64] on the management of tuberculosis in children and adolescents are valuable resources for further reading.

Children affected by RR-TB are one of the most neglected groups with TB. The global TB community must advocate for more and faster research in children on second-line and novel TB drugs and regimens for treatment of RR-TB infection and disease, and increase investment in scaling-up effective approaches, to ensure an equitable response that prioritises the needs of this highly vulnerable population.

## Figures and Tables

**Table 1:** Summary of phase IIc/III clinical studies of adults with MDR-TB or extensively DR-TB^1^ (adapted from [18,24,25]).

Study (N)	Phase	Regimen (Population ^3^)	Outcome	Result
Nix-TB (N = 109) (NCT02333799)	III	6BPaL (XDR-TB ^1^; TI/NR ^2^ MDR-TB)	Relapse-free cure at 12 mo	90% relapse-free cure status 6 mo after end of treatment Led to FDA approval of Pa in the BPaL regimen combination in August 2019 and EMA approval in July 2020
NC-007, ZeNix (N = 181) (NCT03086486)	III	6BPaL1200 2BPaL1200/4BPa 6BPaL600 2BPaL600/4BPa (XDR-TB ^1^; TI/NR ^2^ MDR-TB)	Incidence of bacteriologic failure or relapse/clinical failure through 78 weeks	High rate of favourable clinical outcomes: 93% in 6BPaL1200, 89% in 2BPaL1200/4BPa, 91% in 6BPaL600, and 84% in 2BPaL600/4BPa 6BPaL1200 arm had higher rates of adverse events of peripheral neuropathy and myelosuppression/anaemia
TB- PRACTECAL (N = 630) (NCT02589782)	II/III	6BPaL 6BPaLM 6BPaLC 9–20 mo SOC L600 then 2L300 (MDR-TB; preXDR-TB ^1^; XDR-TB ^1^)	Percentage of patients with unfavourable outcome through 72 weeks	Stage 1 assessed all arms by liquid media culture conversion at 8 weeks. BPaLM advanced to stage 2 In mITT analysis 48.5% (32/66) SOC had an unfavourable outcome vs. 11.3% (7/62) BPaLM Treatment discontinuation more common in control vs. BPaLM 42.4% (28/66) vs. 8.1% (5/62)
NExT (N = 154) (NCT02454205)	III	6–9BLLxTzdZ(Eto or Hhd) 9–20 mo SLID containing reg (MDR-TB)	Treatment success (cure + completion) at 24 mo	Terminated early when South African guidelines changed to replace SLID with BDQ. mITT favourable outcome more than twice as likely in NExT 51% (25/49) vs. control (22.7% 10/44) and faster time to culture conversion
endTB (N = 750) (NCT02754765)	III	9BLMZ 9BLLxCZ 9BLLxDz 9DLLxCZ 9DMCZ 9–20 mo SOC (MDR-TB)	Week 73 Efficacy	Fully enrolled Estimated completion Feb 2023
STREAM II (N = 588) (NCT02409290)	III	4BCLxEZHhdPto/5BCLxEZ 2BCLxZHhdK/4BCLxZ Control (MDR-TB)	Favourable outcome week 76	Fully enrolled Estimated completion Aug 2022
NC-008, SimpliciTB (N = 450) (NCT03338621)	IIc	6BPaMZ (MDR-TB arm)	Time to culture conversion over 8 weeks	Results Q2 2022
BEAT-Tuberculosis (N = 400) (NCT04062201)	III	6BDL (Lx, C or both) 9–12 mo SOC (RR-TB; MDR-TB; FQ-R-RR-TB)	Successful outcome at end of treatment	Recruiting Estimated completion Mar 2023

^1^ Pre-2021 definitions: preXDR-TB = pre-extensively drug-resistant tuberculosis (MDR plus resistance to fluoroquinolone or second-line injectable drug), XDR-TB = extensively drug-resistant tuberculosis (MDR plus resistance to fluoroquinolone and second-line injectable drug); ^2^ Treatment Intolerant/Non-responsive: ^3^ Numbers at the beginning of each regimen represent treatment duration in months, letters represent individual drugs of each regimen. Hd = high-dose, B = bedaquiline, D = delamanid, Pa = pretomanid, L = linezolid, Lx = levofloxacin, M = moxifloxacin, Z = pyrazinamide, Eto = ethionamide, H = isoniazid, Tzd = terizidone (cycloserine), C = clofazimine, SLID = second-line injectable drug, FQ-R-RR-TB = fluoroquinolone-resistant rifampicin-resistant tuberculosis, mo = month/s, wks = weeks.

**Table 2:** Tuberculosis (TB) medications used in children with rifampicin-resistant TB: doses, common adverse effects and comments (adapted from [18,36,37]).

Medication Group & Name	Current Dose Recommendations	Important Adverse Effects	Comments
WHO Group A			
Levofloxacin (Lfx)	15–20 mg/kg/day	Sleep disturbance, GI disturbance, arthralgia/arthritis, idiopathic raised intracranial pressure. Little effect on QT-interval	Modelling data suggest higher doses needed. New dispersible Lfx formulation provides higher exposures than adult formulation [29]
Moxifloxacin (Mfx)	10–15 mg/kg/day	As for levofloxacin, including more QT-interval prolongation effect than Lfx	Pharmacokinetic data in young infants needed. Modelling data suggest higher doses needed
Bedaquiline (Bdq)	>12 years and >30 kg body weight: 400 mg daily x2 weeks, followed by 200 mg M/W/F x22 weeks 6–12 years and 15–30 kg: 200 mg daily x2 weeks followed by 100 mg M/W/F x22 weeks Data on dose in younger children not yet available	Headache, nausea, liver dysfunction, QT-interval prolongation	Dose-finding and safety studies ongoing in children younger than 6 years of age. WHO soon to release interim dosing for children of all ages [10] If used in combination with other QT-prolonging medications, monthly ECG monitoring indicated
Linezolid (Lzd) [28]	Children ≤15 kg body weight: 15 mg/kg once daily Children/adolescents >15 kg 10–12 mg/kg once daily	Diarrhoea, headache, nausea, myelosuppression, peripheral neuritis, optic neuritis, lactic acidosis and pancreatitis	Complete blood count and differential white cell counts to be done 2-weekly for first month, then monthly. Monitoring for vision and peripheral neuritis also important. Adverse effects common
WHO Group B			
Clofazimine (Cfz)	2–5 mg/kg/day. Because of current 50 or 100 mg gel capsule/tablet formulations, alternative day dosing may be necessary in young children. (long half-life)	Skin discolouration, ichthyosis, QT-interval prolongation, abdominal pain.	Pharmacokinetic studies in children ongoing. Child-friendly dispersible tablets available
Cycloserine (Cs) /Terizidone (Tzd)	15–20 mg/kg/day	Neurological and psychological adverse effects	Pharmacokinetic studies in children ongoing
WHO Group C			
Ethambutol	15–25 mg/kg/day	Optic neuritis	Only to use in longer MDR-TB regimen if susceptibility is confirmed
Pyrazinamide	30–40 mg/kg/day	Arthritis/arthralgia (especially with fluoroquinolone use), hepatitis, skin rashes	Only to use in longer MDR-TB regimen if susceptibility is confirmed
Amikacin * (Am) or Streptomycin (Sm)	15–20 mg/kg IMI or IVI daily	Ototoxicity (irreversible), nephrotoxicity	Higher doses only if therapeutic drug monitoring (TDM) is available. To use only if confirmed susceptibility as part of salvage regimens Kanamycin and capreomycin NOT recommended
Delamanid (Dlm)	>12 years/≥35 kg: 100 mg twice daily 6–12 years />20–34 kg: 50 mg twice daily 3–5 years/10–20 kg: 25 mg twice daily Data in younger children not yet available	Nausea, vomiting, dizziness, paraesthesia, anxiety, QT-interval prolongation, hallucinations and night terrors	Dose-finding and safety studies ongoing. WHO soon to release interim dosing for children of all ages—doses in older children may be adapted [28] For pretomanid (Pa), a similar novel agent, no pharmacokinetic data available in children
Meropenem (Mpm)	20–40 mg/kg 8 hourly (IV)	GI intolerance, hypersensitivity reactions, seizures, liver and renal dysfunction	Always combine with clavulanate (Amoxiclav)
Amoxicillin-clavulanate (Amx-Clv)	75 mg/kg/day in 3 divided doses of amoxicillin component	GI intolerance, hypersensitivity reactions, seizures, liver and renal dysfunction	Always combine with a carbapenem (not effective on its own)
Ethionamide (Eto) /Prothionamide (Pto)	15–20 mg/kg/day	GI intolerance, metallic taste, hypothyroidism. Rare: gynecomastia	Co-resistance if *inhA* promoter region mutation confers isoniazid resistance
*Para*-aminosalicylic acid (PAS)	200–300 mg/kg daily as single dose (only divide dose if single dose not tolerated)	GI intolerance, hypothyroidism, hepatitis	Pharmacokinetic studies ongoing. Tolerance with single daily dose good according to experience
Other medications not in WHO groups			
Isoniazid (H) high-dose ^#^	15–20 mg/kg/day (maximum 400 mg)	Hepatitis, peripheral neuropathy	High-dose H had good early bactericidal activity in adults with MDR-TB with *inhA* mutation [38]

^#^ If Isoniazid is used, supplement with pyridoxine (Vitamin B6) in infants and adolescents, in all malnourished and HIV-positive children, and when high-dose isoniazid is used to prevent peripheral neuropathy. * Can be given with lidocaine to reduce the pain of IM injections. GI = gastrointestinal; M/W/F = Monday/Wednesday/Friday.

## Data Availability

Not applicable.
